# A Comparative Study on Pain Perception in Children, After Application of Pre-Cooled and Plain Topical Anaesthetic Gel During Local Anaesthetic Administration—A Parallel Three-Arm Randomised Control Trial

**DOI:** 10.3390/children12070863

**Published:** 2025-06-30

**Authors:** Prabhadevi C. Maganur, Atiah Abdulrahman Ghawi, Ghadi DuhDuh Arishi, Hammam Ahmed Bahammam, Noura Alessa, Nebras Essam Hamed, Nada Ali Jawhali, Mohammed Sawady, Asim Ibrahim H. Manqari, Satish Vishwanathaiah

**Affiliations:** 1 Division of Pediatric Dentistry, Department of Preventive Dental Sciences, College of Dentistry, Jazan University, Jazan 45142, Saudi Arabia; cgowda@jazanu.edu.sa; 2 Dental Intern, College of Dentistry, Jazan University, Jazan 45142, Saudi Arabia; 201805588@stu.jazanu.edu.sa (A.A.G.); 201910152@stu.jazanu.edu.sa (N.E.H.); 201908847@stu.jazanu.edu.sa (N.A.J.); 3 General Dentist, Ministry of Health, Riyadh 11176, Saudi Arabia; gdarishi@moh.gov.sa; 4 Department of Pediatric Dentistry, Faculty of Dentistry, King Abdulaziz University, Jeddah 21589, Saudi Arabia; habahammam@kau.edu.sa; 5 Department of Pediatric Dentistry and Orthodontics, Dental College, King Saud University, Riyadh 11421, Saudi Arabia; nalessa@ksu.edu.sa; 6 Division of Orthodontics, Department of Preventive Dental Sciences, College of Dentistry, Jazan University, Jazan 45142, Saudi Arabia; mesawady@jazanu.edu.sa; 7 General Dentist, Jazan 45142, Saudi Arabia; asim.mongare@hotmail.com

**Keywords:** ice pack, local anaesthesia, pre-cooled gel, pain perception, topical anaesthesia, child, cold temperature

## Abstract

**Background:** Effective pain management in children is essential, particularly when administering local anaesthesia. This study was undertaken to compare pain perception in children after application of pre-cooled and plain topical anaesthetic gel during local anaesthetic administration. **Methods**: A randomised, single-blinded controlled trial was conducted among 51 children between the ages of 6 and 12, visiting the paediatric clinic, Jazan (REC-45/10/1070). Children were allocated into one of the following three groups using a simple randomisation having a 1:1:1 allocation ratio into Group I (*n* = 17): Plain topical anaesthetic gel, Group II (*n* = 17): Pre-Cooled topical anaesthetic gel, and Group III (*n* = 17). An ice pack was applied for a period of 1 min at the injection site. The intensity of pain and the behaviour of the children were assessed using Face, Leg, Activity, Cry, Consolability (FLACC), the Modified Wong–Baker Scale (WBS) and the Frankel Behaviour Rating Scale (FBRS). **Results**: A significant difference in FBRS scores was observed during anaesthesia, with the highest median score [3 (3,3)] in the pre-cooled topical anaesthetic gel group (*p* value < 0.001). FLACC scores varied significantly among groups, with the ice pack group [3 (3, 3)] and [4 (4, 5)] showing the highest median score (*p* value < 0.001). WBS scores also differed significantly between groups (*p* value < 0.001) with a lower value in the pre-cooled topical gel group [0 (0, 0), 2 (0, 2)]. **Conclusions**: This study concluded that, the use of a pre-cooled topical anaesthetic gel before LA administration reduced the pain better than that of plain anaesthetic gel and ice pack application at the injection site during infiltration.

## 1. Introduction

Experiences of pain in childhood could shape one’s understanding as well as perception of pain as an adult [1]. Therefore, effective pain management in children is not only crucial but a challenging task in paediatric dentistry [2]. A child’s limited ability to cooperate due to fear and anxiety are closely linked to past negative dental experiences, familial attitudes, facial trauma, heightened emotional sensitivity and low pain tolerance [3]. Along with these psychological factors, physical challenges, such as dealing with small oral structures as well as addressing developmental changes in child’s teeth, can greatly hinder the delivery of high quality dental care [4]. Pain management in dentistry requires comprehensive and sensitive approaches. The alleviation of pain in dentistry includes various methods, such as pharmacological and non-pharmacological techniques. The administration of anaesthetic agents and usage of procedures to reduce pain before a surgical intervention are included in pre-emptive methods of pain management [5].

The usage of needles and syringes for pain control has a negative impact on the child due to the fear and anxiety associated with it [6]. Postponement of dental visits is attributed to this fear of needles and biting injury from injection among the paediatric population [6]. To alleviate anxiety and to achieve adequate anaesthetic effect, numerous techniques have been used recently for pain control in dentistry [7]. These include the application of topical anaesthetic agents, changing the temperature of local anaesthesia (LA) agents, buffering LA, adjusting the rate of infiltration by reducing the speed of injection, distraction techniques, vibrating the surrounding tissue while injecting the agent, applying pressure at the injection site, counter irritation, computer-controlled delivery techniques, and usage of tools, such as vibrajet and dental vibe [7]. Alternative delivery systems, such as the use of electronic dental anaesthesia, jet injectors, iontophoresis and computer-controlled local anaesthesia, have also been reported [8,9].

As LA administration via injection is the most common method used, several measures to reduce the pain caused during needle penetration have been reported [10]. Topical gel application is the conventional method used before LA administration [10]. The numbing effect created by topical gel application depends on various factors, such as the pace used for injection and the gauge size of the needle used [10]. This method is not always reported as a pain-free injection technique [10]. Also, the duration of action of the anaesthetic effect created by topical gel varies from 5–10 min along with other disadvantages of unpleasant taste and spreading of anaesthetic agent to non-injection sites [11]. In this regard, innovative techniques using cryoanaesthesia are gaining traction as novel approaches [12].

Controlled localised application of cold to block the conduction of nerve signals through the inhibitory pain regulating gate control system at the spinal cord level is referred to as cryoanaesthesia [12]. Neuropraxia created using cold decreases the velocity of pain signal conduction and also the threshold required for activation of nociceptors [12]. Cold application is used to relieve pain during sports injuries, burns, sprains, fractures, bruises and insect bites. Although cooling the tissue has a deep-rooted history in medicinal practice [13], it has not been used routinely in dentistry [10]. The usage of ice for cooling the injection site has been shown to reduce oedema, nerve conduction velocities, cellular metabolism and blood flow to the area [4].

Cooling the topical anaesthetic gel and cooling the injection site are both different forms of cold application techniques in pain control [2,14]. A systematic review by Tirupathi SP et al. suggested that injection site pre-cooling using ice could be a useful supplement to topical anaesthesia for lowering both subjective as well as objective pain. But it also recommended that further studies with better designs are required for more conclusive results about its effectiveness [15]. Previous studies have analysed the effectiveness of pre-cooling the injection site with various forms of topical anaesthesia techniques such as use of pre-cooled gel and use of ice for pre-cooling separately [2,14,16,17,18]. There is a scarcity in literature comparing the effect of both pre-cooling the site and cooling the topical anaesthetic gel prior to LA administration. Hence, a study was done with an objective to assess and evaluate the effect of pre-cooling the injection site and the usage of cooled topical anaesthetic gel and plain topical anaesthetic gel prior to LA administration on pain perception among children. The null hypothesis stated that, there is no difference in the intensity of pain perceived while using an ice pack prior to LA administration, plain topical anaesthesia gel and pre-cooled topical anaesthesia gel prior to LA administration.

## 2. Materials and Methods

### 2.1. Study Design, Ethical Clearance and Informed Consent

The current study is a randomised, parallel three-arm, single-blinded controlled trial that was reviewed and approved by the institutional human ethical committee with registration number REC-45/10/1070. This research followed the guidelines of the Consolidated Standards of Reporting Trials (CONSORT) to ensure transparency and validity in reporting. The study design and procedures were carefully planned and conducted in accordance with ethical standards to maintain the integrity and quality of the investigation. Prior to participant enrolment, the detailed protocol, inclusive of specific details, was presented to the parent or legal guardian. Only after securing a signed written informed consent were the participants admitted to the study. Adherence to the ethical standards set forth in the Declaration of Helsinki (1964) and its subsequent revisions was strictly followed in conducting this study.

### 2.2. Sample Size Estimation

Based on proportional differences observed in a prior study [2], where 60% of children experienced significant pain during plain LA insertion (measured using visual analogue scale) versus 13.3% with pre-cooled LA, the first sample size estimate was made. A sample size of 13 participants per group was established using an absolute difference of 46.7%, a 95% confidence level (α = 0.05), and a power of 80% (β = 0.20). In order to account for possible attrition, this calculation was then inflated by 20% to 17 per group.

### 2.3. Participant Selection

#### 2.3.1. Inclusion Criteria

Children between the ages of six and twelve who required LA administration (infiltration) in either the maxillary arch or mandibular arch were considered. The study included children who had favourable or certainly positive conduct during the pre-treatment evaluation (scores of 3 or 4) on the Frankel scale, which means the children have positive acceptance of treatment, at times cautious, willingness to comply with the dentist, at times with reservation but patient follows the dentist’s directions cooperatively or definitely positive, good rapport with the dentist, interested in the dental procedures, laughing and enjoying the situation.

#### 2.3.2. Exclusion Criteria

Children with systemic sickness (American Society of Anaesthesiologist’s ASA II and III), a known allergy to local anaesthetics or who required emergency dental care were excluded.

### 2.4. Study Sample

Study sample constituted 51 children between the ages of 6 and 12, who were scheduled to undergo any three simple routine dental procedures, such as restorations or extractions (Grade III mobility), that necessitated the use of local infiltration while visiting the paediatric dentistry clinic, Jazan University, Jazan, Saudi Arabia.

### 2.5. Randomisation and Allocation Concealment

**Figure 1:**
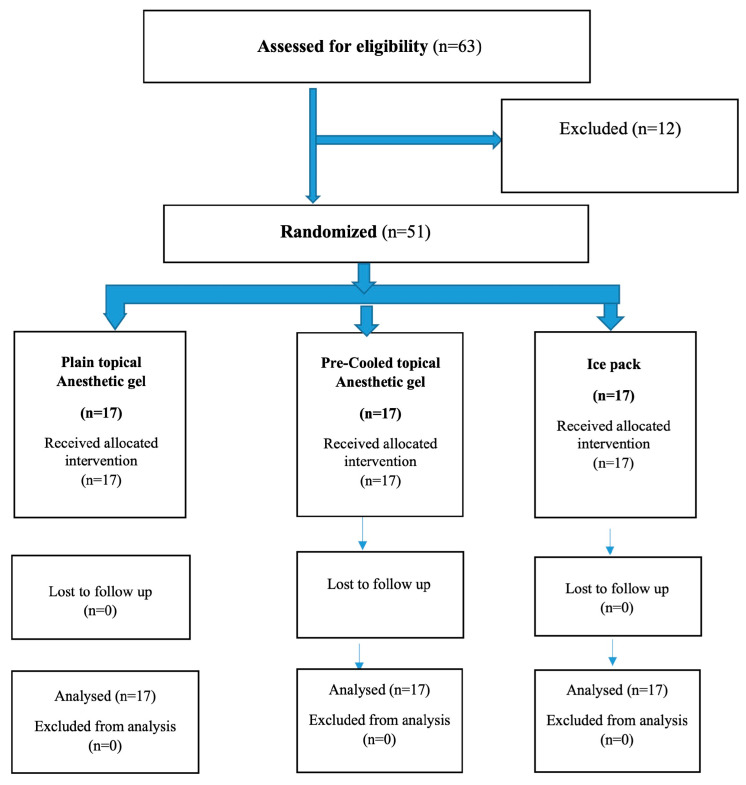
Schematic representation of the study design (CONSORT Flow).

Children were randomly assigned to one of two groups utilising the SNOSE (sequentially numbered, opaque, sealed envelopes) allocation concealment method at a 1:1:1 allocation ratio. A staff member independent of the recruitment and evaluation team prepared and maintained the envelopes and randomisation code. To ensure impartial distribution, envelopes were opened one after the other during the intervention. The evaluator who assessed pain scores was blinded to group assignments. The study groups were as follows [Figure 1]: Group I (*n* = 17) received plain topical anaesthetic gel; Group II (*n* = 17) received pre-cooled topical anaesthetic gel; and Group III (*n* = 17) received an ice pack for 1 min at the injection site. The temperature of pre-cooled anaesthetic gel was maintained at 4 °C using a normal medicine refrigerator.

### 2.6. Procedure

***Stage I***: Baseline assessment: Following the patient’s seating on the dental chair, prior to any interventions, the pulse rate was assessed utilising a pulse oximeter. Five readings were taken every 3 min over a 15-min period, and baseline readings were recorded. The initial readings provide baseline data for the objective assessment of the patient’s condition during the appointment. The patient’s behaviour was evaluated using the Frankel behaviour rating scale.

**Figure 2:**
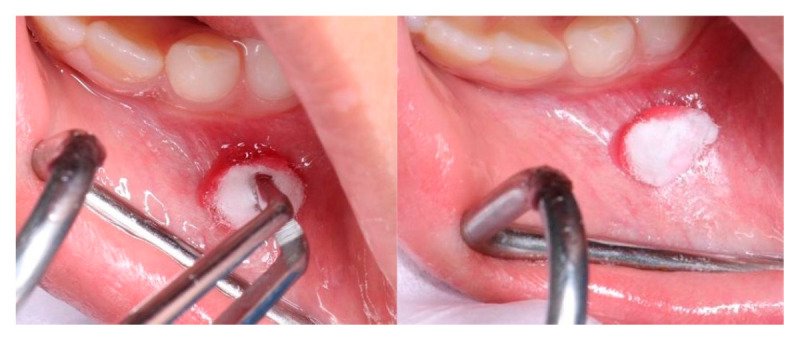
Group I and II.

**Figure 3:**
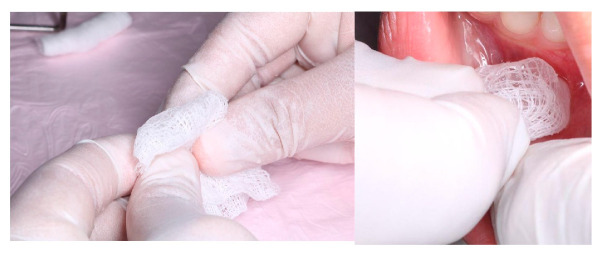
Group III.

***Stage II***: The site where local anaesthesia was to be administered was first identified, then cleaned with a sterile piece of gauze, and dried. Following that, the appropriate topical agent was applied using a cotton-tipped applicator. After 1 min of applying the topical gel, local anaesthesia was administered in groups I and II [Figure 2]. In group III, an ice stick covered in gauze was placed on the site for 1 min before administering the local anaesthesia [Figure 3]. The patient’s pulse rate was monitored during the needle insertion. Additionally, the patient’s behaviour was assessed using a behaviour rating scale, and pain levels were evaluated using the FLACC and Wong–Baker Pain Scales.

***Stage III***: Once the baseline reading was achieved on the pulse oximeter, the local anaesthetic solution was administered. The time taken for the administration of the local anaesthetic was recorded in seconds. During the procedure, the FLACC and behaviour rating scale were assessed. A highest post-injection pulse rate was noted. Once the pulse rate returned to the baseline after a minute, the patient was asked to complete the Wong–Baker Scale to evaluate subjective pain perception. The behaviour rating scale was also utilised during this stage.

***Stage IV***: Following treatment completion, the pulse rate was documented again. The patient was discharged once the initial readings were achieved. Postoperative instructions were verbally provided to the parents and child. Post-treatment assessments using the Wong–Baker Scale and behaviour rating scale were conducted. The second intervention was performed one week after the initial procedure. A single operator carried out the procedure for all subjects throughout the study, and a single examiner measured the parameters to rule out any operator bias in recording the scores.

### 2.7. Statistical Analysis

Statistical analysis was carried out using a standard statistical package (SPSS 20 for Windows, SPSS Inc., Chicago, IL, USA). The normality of data was assessed using a Shapiro–Wilk test [Supplementary Table]. The distribution of participants to the two groups based on the age and gender was analysed using the Chi-square test. The intergroup comparisons of metric and ordinal data were carried out using one-way ANOVA and the Kruskal–Wallis test, respectively. Friedmann multiple post hoc comparison was performed to analyse FBRS and WBS scores at different time periods within each group.

### 2.8. Outcomes

One investigator assessed the primary outcomes, including the Face, Legs, Activity, Cry, Consolability (FLACC) Scale [19] and the Wong–Baker Faces Rating Pain Scale (WBS) [20]. Another investigator evaluated the secondary outcomes, which comprised the time required to administer anaesthesia, the Frankel Behaviour Rating Scale (FBRS) [21] and pulse rate.

#### 2.8.1. Primary Outcomes

1. Face, Legs, Activity, Cry, Consolability (FLACC) Scale

During the administration of the local anaesthetic, the FLACC Scale was utilised for the objective of pain assessment. To measure how severe the pain is, the FLACC Scale looks at five distinct domains of behaviour. Checks the face for signs of distress, such as frowns and grimaces. Legs as a whole measure nervousness, agitation or stiffness. Overall bodily activity, restlessness or incapacity to remain motionless are assessed by activity. Cry evaluates vocal expressions of pain, like crying or vocalisations. Consolability measures how easily the child can be comforted. Each domain is given a score of 0–2, with 0 indicating no pain and 2 indicating the highest pain. The sum of the points in each domain gives a total score that can be anywhere from zero to ten. A lower score denotes less intense pain, whereas a higher value implies more severe pain [19].

2. Wong–Baker Scale

In this study, the pain assessment tool used was the Wong–Baker Facial Expressions Rating Scale. Each of the six facial expressions on this scale has a numerical value between zero and ten that represents the degree of pain that the user is experiencing. The scores on the scale were used to classify the levels of pain; mild pain was represented by values between 0 and 4, moderate pain by values between 4 and 6, severe pain by values between 6 and 8, and excruciating pain by values between 8 and 10. At four intervals, children in both groups were asked to rate their pain using the Wong–Baker Pain Scale by choosing the most suitable statement: immediately after topical administration, prior to the injection, immediately after the injection, during the treatment, and after the treatment [20].

#### 2.8.2. Secondary Outcome

1. Local anaesthetic administration time:

The duration of time required to administer the local anaesthetic solution was noted. The total time from the application of the topical anaesthetic/ice pack to its removal from the participant’s mouth was measured by a third investigator using a stopwatch.

2. Frankl Behaviour Rating Scale

The child’s behaviour during different stages of a dental procedure was assessed using Wright’s modification of FBRS, a commonly used scale to evaluate a child’s cooperation and response during dental procedures. Behaviour was evaluated throughout the procedure, including pre-procedure assessment of the FBRS during the application of topical medication, the examination and radiography stages. The FBRS was monitored during the administration of local anaesthesia within the procedure. Post-procedure, the FBRS was observed both during the procedure and as the patient left the dental chair [21].

3. Pulse rate

To account for the potential rise in pulse rate caused by stress or anxiety, a pulse oximeter device (Dr. Trust Pulse Oximeter, Nureca Limited, India) was placed on the left index finger in order to record pulse rate readings. This metric was utilised as an indicator of anxiety. Pulse rate readings were obtained every 3 min before the procedure, yielding a total of five readings within a 15-min period: the initial reading at 0 min, followed by subsequent readings at 3, 6, 9 and 12 min. The pulse rate was then monitored during the application of topical anaesthesia, with a dedicated reading during the anaesthesia process. Additionally, a pulse rate measurement was taken immediately after local anaesthesia administration. These supplementary outcomes were monitored by an unbiased individual not involved in the study, maintaining the blinding procedure for the anaesthesia protocol.

## 3. Results

The distribution of the participants to the three groups in relation to age and gender is described in Table 1.

Statistically significant difference was not observed (*p* value > 0.05) across three groups for age, time of LA administration and pulse rate during anaesthesia as shown in Table 2.

While looking at the ordinal characteristics, we found that the FBRS score during anaesthesia showed very high statistically significant differences (*p* value < 0.001), with the pre-cooled topical anaesthetic gel group having the highest median score of 3. Analysing FLACC scores during topical and LA administration revealed a very high statistically significant difference (*p* value < 0.001) among the three groups, and the median FLACC score in the ice pack group was [3 (3, 3)] and [4 (4, 5)] found to be the highest. A very high statistically significant difference (*p* value < 0.001) was obtained for WBS scores immediately after topical anaesthesia and after LA injection, with a lesser value in the pre-cooled topical anaesthetic gel group [0 (0, 0), 2 (0, 2)]. A statistically significant difference (*p* value < 0.01) was also obtained at the end of treatment comparison for WBS scores, as shown in Table 3.

It was observed that all three groups showed statistically significant differences in FBRS scores across the time points (before, during, and after anaesthesia), as indicated by *p*-values less than 0.05. Friedmann post hoc comparison revealed that Groups II and III experienced a statistically significant change in behaviour from before to during anaesthesia, as described in Table 4 and Table 5.

Similarly, pain levels varied significantly during the anaesthesia process for all three groups (*p* value = 0.001). Groups II and III showed multiple significant differences, especially in comparisons involving immediate post-injection pain, as described in Table 6 and Table 7.

## 4. Discussion

Local anaesthesia is instrumental in ensuring patient comfort during procedures in paediatric dentistry, but it is essential to acknowledge the anxiety that often accompanies it [11]. Hence, measures to reduce the discomfort and apprehension associated with its administration are essential [11]. Topical anaesthesia has been a frequently employed method to ease discomfort during the administration of local anaesthesia, especially for superficial procedures or prior to injections [14]. Cryoanaesthesia is an emerging promising technique that can be used along with LA administration but its application in paediatric dentistry is still being explored. Hence, a study was conducted to evaluate the effect of various forms of cryoanaesthesia methods with plain topical anaesthetic gel prior to LA administration on pain perception among children.

Cryoanaesthesia causes activation of A delta fibres, which inhibits the pain pathway, leading to an increased pain threshold [22]. This cold-induced neuropraxia could lead to reduced blood flow, resulting in vasoconstriction, decreased oxygen utilisation and inflammation [22]. This could be achieved through various methods, such as cooling the tissue site or cooling the anaesthetic agent used for anaesthesia, usage of refrigerant sprays or ice packs [23]. By cooling the tissues locally, neuromuscular transmission is delayed, and pain signal conduction is halted [10]. This could also result in decreased muscular tone due to the reduced spindle activity in the muscles [10]. A study by Bose et al. suggested that a one minute application of ice could reduce the pain perception, irrespective of the type of anaesthesia used [24].

Present study assessed the effectiveness of cooling the tissue site as well as cooling the anaesthetic gel before the LA administration. Although the discomfort associated with LA administration has been reported to be lesser in the pre-cooled topical anaesthetic gel group, when compared to the other groups, there was no difference in the time required for LA administration between the groups in the current study. This could be attributed to the standardised techniques used in the study for LA administration and the calibration of the examiner. Lesser discomfort during the procedure reflected the reduced anxiety experienced by these children in the present study.

A key contributing factor to the anxiety in paediatric dental visits is local anaesthesia administration. Anxiety level measurement could be achieved through various direct and indirect techniques [25]. The pulse rate measured through palpation could be used as an objective metric for gauging anxiety in an individual [25]. An elevated pulse rate could be a critical physiological indicator of anxiety, particularly in children, where assessing other responses could be challenging [25]. Although there was no statistically significant difference between the groups in the mean pulse rate measured, the value was reduced in the pre-cooled anaesthetic gel group one minute after LA administration and during anaesthesia when compared to that of mean pulse rate assessed before anaesthesia in the present study. This was in line with the study conducted by Chilakamuri et al., who showed no significant change in the pulse rate among individuals administered an ice pack and plain lignocaine gel during anaesthesia [26]. This could be due to the cooling effect of ice or the anaesthetic effect of the gel, which might not have been long enough to assess a measurable physiological response, such as a significant change in pulse rate. A contradictory finding was reported by Virdikar et al., who found that individuals treated with pre-cooled anaesthetic gel before LA administration showed a lower mean pulse rate when compared to that of those treated with plain lignocaine gel before LA administration [2].

Association between anxiety and behaviour has been well established, emphasising the importance of evaluating behaviour to pinpoint those requiring extra attention during dental appointments [27]. Among the different behaviour rating scales, FBRS is considered as the most reliable tool in paediatric research as well as daily clinical practice [28]. A definitely positive behaviour was shown by the children in all three groups in the present study before LA administration. But later, during LA administration, there was a change in behaviour from definitely positive to either positive or negative in all three groups in the current study. This could be due to the anxiety these children had regardless of the method used. During anaesthesia administration, the children in the pre-cooled topical anaesthetic gel group showed a positive behaviour score when compared to other groups. This significant difference in score in the current study might be due to the reduced discomfort caused by the pre-cooled anaesthetic gel when compared to that of plain gel and ice pack application at the injection site. Children with a Frankel’s Behaviour Rating Scale III and IV were only included in previous studies by Virdikar et al. and Nat et al. [2,10].

Emotional and situational distress hinders the child’s ability to self-report their feelings [26]. Therefore, an objective tool to assess the pain and distress experienced by the child could add to the accuracy of evaluation, providing a better understanding of the child’s behaviour [26]. Our study employed the FLACC Scale to fulfil this purpose, offering a reliable and systematic method for assessing the child’s discomfort and distress while using different techniques for pain control. Changes in the facial expression, body activity and crying could suggest important information regarding the comfort and anxiety experienced by the child. FLACC scores among children treated with pre-cooled anaesthetic gel, ice pack application and plain topical anaesthetic gel showed very high statistically significant differences in the present study. Lower FLACC scores suggested lesser anxiety and enhanced patient comfort. The pre-cooled anaesthetic gel group showed lesser FLACC scores in the current study compared to other groups. Studies by Chilkamuri et al. and Dhingra et al. reported lesser FLACC scores in participants treated with ice cubes before LA administration than those treated with plain topical anaesthetic [26,29].

Assessment of pain experienced by the children during the procedures was done using the Wong–Baker Faces Pain Rating Scale. This is a subjective scale that is used by children to record the pain they experienced during the procedures. Pain scores reported by the children immediately after injection in the pre-cooled anaesthetic gel group was lower when compared to that of the scores reported by the ice pack application group and plain anaesthetic gel group in the present study and the differences between the groups were statistically highly significant. A study by Anantharaj A et al. reported no significant difference in the pain scores between plain anaesthetic gel, the ice-cooled injection site group and the clove papaya group [14]. The least pain reduction was reported by the topical anaesthesia group in a study by Padmanabhan V et al., followed by pre-cooling the site and the vibration group, which was in accordance with this study [16]. Pre-cooling the site before LA administration has been reported to show the lowest pain score in previous studies when compared to other techniques, like plain topical anaesthetic gel, buffered local anaesthesia and vibration [6,30].

Other pain and discomfort assessment scales used in previous studies are VAS (Visual Analog Scale), Coloured Analogue Scale and SEM (Sound, Eye, Motor Scale) [6,10,22]. Mean VAS scores of children in the pre-cooled anaesthetic gel group were lower compared to the plain anaesthetic gel group in a study by Nat et al. [10]. Pre-cooling the injection site before LA administration has been reported to result in less discomfort for the participants in previous studies [23,26,30,31].

The anaesthetic effect of the topical anaesthetic gel arises from its ability to induce analgesia by penetrating the mucosal lining [18]. By blocking the signal conduction from the nerve terminal, it creates surface anaesthesia to a depth of 2–4 mm [18]. When this topical gel was used after pre-cooling, the added benefit of cryoanaesthesia, such as neuropraxia, could have enhanced the analgesic effect of the gel. This could be the reason for the better comfort reported by the children in the pre-cooled topical anaesthetic gel group when compared to that of the other groups with LA administration in the present study.

To the best of our knowledge, this was the first study that explored the difference in pain experienced by the children that employed pre-cooling the injection site using ice, pre-cooling the anaesthetic gel and plain anaesthetic gel. A thorough evaluation of pain using both subjective and objective measurement scales along with physiological indicators was done in the study. Moreover, the study’s design and the application of standardisation protocols could add to its strength and credibility. The inclusion of a smaller number of participants in each group could restrict the generalisability of the results. The absence of dental anxiety evaluation, which has a substantial impact on a child’s perception of pain, can be considered as another drawback. Validated anxiety measures should be used in future research to better account for this variable and offer a more thorough knowledge of how pain is modulated during the administration of local anaesthetics. In addition to it, the sample size was determined using binary proportions from a previous two-group based investigation to detect all pairwise group differences in a three-arm randomised design. Despite offering enough power for primary comparisons, this could have diminished the ability to identify minute variations among all three groups in the present study. A prior sample size estimates that specific multiple-group comparisons should be taken into account in future research to guarantee sufficient power for identifying pairwise differences.

## 5. Conclusions

This study found that applying a pre-cooled topical anaesthetic gel prior to administering LA was more effective at reducing pain than using a regular anaesthetic gel and applying an ice pack at the injection site during infiltration. Compared to the other groups, the kids in the pre-cooled topical anaesthetic gel group behaved better overall.

## Figures and Tables

**Table 1:** Distribution of study participants in relation to age and gender.

SI. No	Parameters	Plain Topical Anaesthetic Gel N (%)	Pre-Cooled Topical Anaesthetic Gel N (%)	Ice Pack Application for a Period of 1 Min at the Injection Site N (%)	*p* Value
1	Age			10 (58.8)	1
	6–9 years	10 (58.8)	10 (58.8)		
	10–12 years	07 (41.1)	07 (41.1)	07 (41.1)	
2	Gender	11 (64.7)	11 (64.7)	11 (64.7)	
	Male				
	Female	06 (35.3)	06 (35.3)	06 (35.3)	1

**Table 2:** Comparison of various parameters (metric data) during local anaesthesia administration.

SI. No	Parameters	Plain Topical Anaesthetic Gel (Mean ± SD)	Pre-Cooled Topical Anaesthetic Gel (Mean ± SD)	Ice Pack Application for a Period of 1 Min at the Injection Site (Mean ± SD)	*p* Value
1	Time required for administration of LA in seconds	40.23 ± 12.10	42.40 ± 6.02	43.29 ± 5.19	0.551
2	**Pulse rate** Before anaesthesia	91.31 ± 10.80	96.14 ± 10.20	88.76 ± 9.21	0.202
	During topical anaesthesia	93.58 ± 12.28	95.76 ± 9.23	87.47 ± 16.89	0.175
	During anaesthesia	97.23 ± 10.98	97.80 ± 8.80	93.29 ± 12.50	0.420
	1 min after LA administration	91.70 ± 12.41	93.23 ± 8.70	89.58 ± 10.45	0.607

**Table 3:** Comparison of various parameters (ordinal data) during local anaesthesia administration.

SI. No	Parameters	Plain Topical Anaesthetic Gel [Median (Q1, Q3)]	Pre-Cooled Topical Anaesthetic Gel [Median (Q1, Q3)]	Ice Pack Application for a Period of 1 Min at the Injection Site [Median (Q1, Q3)]	*p* Value
1	FBRS Before anaesthesia	4 (3, 4)	4 (3, 4)	4 (4, 4)	0.546
	During anaesthesia	2 (2, 4)	3 (3, 3)	2 (2, 4)	<0.001 ***
	After anaesthesia	3 (3, 4)	3 (3, 4)	3 (3, 4)	0.781
2	FLACC Scale during topical anaesthesia	0 (0, 0)	1 (0, 2)	3 (3, 3)	<0.001 ***
3	FLACC Scale during LA	3 (2, 3)	1 (0, 1)	4 (4, 5)	<0.001 ***
4	WBS Immediately after topical anaesthesia	0 (0, 0)	0 (0, 0)	0 (0, 2)	<0.001 ***
	Immediately after injection	4 (4, 6)	2 (0, 2)	6 (4, 8)	<0.001 ***
	Before the procedure	2 (0, 2)	0 (0, 0)	2 (0, 2)	0.0531
	During the treatment	0 (0, 0)	0 (0, 0)	0 (0, 2)	0.0605
	At the end of the treatment	0 (0, 0)	0 (0, 0)	0 (0, 0)	0.01 **

** Significant at 0.01 level, *** Significant at 0.001 level. FBRS = Frankel Behaviour Rating Scale, FLACC = Face, Legs, Activity, Cry, Consolability, WBS = Wong–Baker Scale.

**Table 4:** Comparison of FBRS at different time periods within each group using the Friedmann test.

FBRS		Before, During and After Anaesthesia		
		Group I	Group II	Group III
Friedmann test value		15.08	16.47	12.09
*p* value		0.001 *	0.001 *	0.002 *
95% CI (Lower, Upper)	Before	(3.39, 3.90)	(3.54, 3.99)	(3.33, 3.84)
	During	(2.23, 3.18)	(2.10, 3.19)	(2.81, 3.42)
	After	(2.68, 3.66)	(3.16, 3.67)	(3.21, 3.73)
	Effect size	0.19	0.31	0.13

* Significant.

**Table 5:** Friedmann post hoc comparison of FBRS at two time intervals for Groups I, II and III.

Pair Wise Comparison- Post Hoc FBRS	Group I		Group II		Group III	
	Test Statistic	Adj. Sig. *p* Value	Test Statistic	Adj. Sig. *p* Value	Test Statistic	Adj. Sig. *p* Value
During anaesthesia vs. After anaesthesia	−0.50	0.43	−0.62	0.22	−0.64	0.178
During anaesthesia vs. Before anaesthesia	0.645	0.18	0.97	0.01 *	1.03	0.01 *
After anaesthesia vs. Before anaesthesia	0.145	1.00	0.35	0.91	0.38	0.79

Bonferroni adjustment, * Significant.

**Table 6:** Comparison of WBS during different time periods within each group using the Friedmann test.

WBS		Immediate After Topical, Injection, Before, During and at the End		
		Group I	Group II	Group III
Friedmann test value		41.82	30.93	47.85
*p* value		0.001 *	0.001 *	0.001 *
95% CI (Lower, Upper)	Immediate	(0.41, 1.47)	(0.02, 0.92)	(0.00, 0.00)
	After the injection	(4.65, 7.11)	(0.89, 2.39)	(3.29, 5.65)
	Before	(0.67, 1.92)	(−0.11, 1.05)	(0.62, 2.21)
	During	(0.08, 1.33)	(0.00, 0.00)	(−0.05, 0.76)
	At the end	(0.02, 0.92)	(0.00, 0.00)	(0.00, 0.00)
Effect size		0.68	0.32	0.65

* Significant.

**Table 7:** Friedmann post hoc comparison of WBS at two time intervals for Groups I, II and III.

Pair Wise Comparison- Post Hoc WBS	Group I		Group II		Group III	
	Test Statistic	Adj. Sig. *p* Value	Test Statistic	Adj. Sig. *p* Value	Test Statistic	Adj. Sig. *p* Value
Immediately after topical anaesthesia vs. At the end of the treatment	0.50	1.00	0.00	1.00	0.18	1.00
Immediately after topical anaesthesia vs. During the treatment	0.50	1.00	−0.35	1.00	0.44	1.00
Immediately after topical anaesthesia vs. Before the procedure	1.70	0.02	−1.12	0.39	0.79	1.00
Immediately after topical anaesthesia vs. Immediately after injection	1.70	0.02	−2.50	0.001 *	2.56	0.001 *
At the end of the treatment- vs. During the treatment	0.44	1.00	0.35	1.00	0.26	1.00
At the end of the treatment- vs. Before the procedure	0.44	1.00	1.12	0.39	0.62	1.00
At the end of the treatment vs. Immediately after injection	0.00	1.00	2.50	0.001 *	2.38	0.001 *
During the treatment vs. Before the procedure	0.06	1.00	0.77	1.00	−0.35	1.00
During the treatment-WBS-Immediately after injection	1.26	0.20	2.15	0.001 *	−2.12	0.001 *
Before the procedure vs. Immediately after injection	−1.21	0.26	1.38	0.11	1.76	0.01 *

Bonferroni adjustment, * Significant.

## Data Availability

Data availability made available on request.

## Supplementary Materials

The following supporting information can be downloaded at: https://www.mdpi.com/article/10.3390/children12070863/s1, Table S1: Test of Normality for Metric Data Parameters; Table S2: Test of Normality for Ordinal Data Parameters.

## Abbreviations

The following abbreviations are used in this manuscript:

FLACC	Face, Leg, Activity, Cry, Consolability
WBS	Wong–Baker Scale
FBRS	Frankel Behaviour Rating Scale
LA	Local Anaesthesia
CONSORT	Consolidated Standards of Reporting Trials
SNOSE	Sequentially Numbered, Opaque, Sealed Envelopes
