# Supplementary material for: A Comparative Study on Pain Perception in Children, After Application of Pre-Cooled and Plain Topical Anaesthetic Gel During Local Anaesthetic Administration—A Parallel Three-Arm Randomised Control Trial
Source: Children (Basel). 2025 Jun 30;12(7):863. doi: 10.3390/children12070863 (PMC12293320; doi:10.3390/children12070863)
Supplement: Supplementary file 1 [file children-12-00863-s001.zip › children-3632082-supplementary.pdf]

Table S1 Test of Normality for Metric Data Parameters

Metric Data Parameters	Groups	Shapiro–Wilk		
		Statistic	df	Sig.
Age	Group I	0.88	17	0.06
	Group II	0.88	17	0.06
	Group III	0.88	17	0.06
Pulse rate-Before anaesthesia	Group I	0.95	17	0.43
	Group II	0.94	17	0.36
	Group III	0.95	17	0.43
Pulse rate-During topical anaesthesia	Group I	0.97	17	0.84
	Group II	0.91	17	0.10
	Group III	0.87	17	0.06
Pulse rate-During anaesthesia	Group I	0.96	17	0.55
	Group II	0.90	17	0.07
	Group III	0.91	17	0.09
Pulse rate-1 min after LA administration	Group I	0.95	17	0.41
	Group II	0.97	17	0.80
	Group III	0.94	17	0.35

Shapiro–Wilk test for the metric parameter was found to be statistically not significant; hence, a normal distribution of the data was observed. Therefore, a parametric one-way ANOVA test was used for comparison between the 3 groups with respect to age and pulse rate.

Table S2 Test of Normality for Ordinal Data Parameters

Ordinal Data Parameters	Groups	Shapiro–Wilk		
		Statistic	df	Sig.
FBRS-Before anaesthesia	Group I	0.61	17	0.00
	Group II	0.63	17	0.00
	Group III	0.53	17	0.00
FBRS-During anaesthesia	Group I	0.68	17	0.00
	Group II	0.77	17	0.00
	Group III	0.85	17	0.01
FBRS-After anaesthesia	Group I	0.74	17	0.00
	Group II	0.64	17	0.00
	Group III	0.63	17	0.00
FLACC-During topical anaesthesia	Group I	0.00	17	0.00
	Group II	0.53	17	0.00
	Group III	0.00	17	0.00
FLACC-During LA	Group I	0.58	17	0.00
	Group II	0.39	17	0.00
	Group III	0.66	17	0.00
WBS-Immediately after topical anaesthesia	Group I	0.00	17	0.00

	**Group II**	0.53	17	0.00
	**Group III**	0.64	17	0.00
**WBS-Immediately after injection**	**Group I**	0.89	17	0.06
	**Group II**	0.81	17	0.00
	**Group III**	0.83	17	0.01
**WBS-Before the procedure**	**Group I**	0.78	17	0.00
	**Group II**	0.49	17	0.00
	**Group III**	0.76	17	0.00
**WBS-During the treatment**	**Group I**	0.47	17	0.00
	**Group II**	0.00	17	0.00
	**Group III**	0.63	17	0.00
**WBS-At the end of the treatment**	**Group I**	0.00	17	0.00
	**Group II**	0.00	17	0.00
	**Group III**	0.53	17	0.00

Shapiro–Wilk test for the ordinal data parameters was found to be statistically significant; hence, a non-normal distribution of the data was observed. Therefore, a non-parametric Kruskal–Wallis test was used for comparison between the 3 groups with respect to FBRS, FLACC and WBS.
